# Excess life-years and productive life-years lost in Poland through the COVID-19 pandemic and post-pandemic years

**DOI:** 10.1186/s13690-025-01792-0

**Published:** 2026-01-15

**Authors:** Błażej Łyszczarz, Jakub Wojtasik, Tomasz Zieliński

**Affiliations:** 1 Department of Health Economics, Nicolaus Copernicus University in Toruń, Bydgoszcz, Poland; 2 Statistical Analysis Centre and Doctoral School of Social Sciences, Nicolaus Copernicus University in Toruń, Toruń, Poland; 3 Statistical Analysis Centre, Nicolaus Copernicus University in Toruń, Toruń, Poland

**Keywords:** COVID-19, Excess mortality, Poland, Years of potential productive life lost, Labour force

## Abstract

**Background:**

Poland’s COVID-19 experience resulted in one of Europe’s most severe mortality shocks, yet the share of lost life-years occurring at working age has not been quantified.

**Methods:**

We analysed weekly all-cause deaths by sex and 5-year age groups for 2011–2019, and fitted fixed effects linear models to forecast expected mortality rates for 2020–2024. Next, we compared predicted and observed rates to estimate excess mortality. These estimates were converted into excess deaths and p-scores, excess Years of Potential Life Lost (eYPLL) and excess Years of Potential Productive Life Lost (eYPPLL).

**Results:**

We estimated 257,246 excess deaths in Poland over the five-year horizon (13.4% and 12.2% more deaths than expected in men and women, respectively). These deaths yielded 1,921,265 eYPLL and 521,887 eYPPLL, meaning that 27% of all life-years lost fell within working age. Men accounted for 54% of excess deaths but 72% of eYPLL and 79% of eYPPLL. More than half of the female and more than a third of the male productive-life loss accrued between ages 30 and 49. Temporal concentration was marked, 46% of excess deaths and 40% of eYPPLL occurred in 2021 alone. In 2023–2024, negative excess mortality emerged among older adults (aged 55–69), but without fully offsetting losses among the working-age and 70 + population. This translated to negative eYPLL in women in 2024, but persistent productive-age losses were still identified in this group. COVID-19-coded fatalities explained almost two-thirds of eYPLL and 40% of eYPPLL during 2020–2024. Poland’s excess mortality resulted in an average loss of 14–15 productive years for every excess death before retirement.

**Conclusions:**

This study extends usual excess deaths estimates to the productive life lost dimension and shows that the pandemic curtailed not only longevity but also burdened Poland with over half a million labour-force years lost. These losses magnify pre-pandemic labour shortages and add demographic pressure, translating to reduced labour supply and potential output, and underscoring the macroeconomic cost of major health shocks such as the COVID-19 pandemic.

**Supplementary Information:**

The online version contains supplementary material available at 10.1186/s13690-025-01792-0.

**Table:** 

Text box 1. Contributions to the literature
• Our study provides first estimates of excess productive life-years lost in Poland during the COVID-19 pandemic and post-pandemic (2020-2024).
• There were 12-13% more deaths than expected in 2020-2024, but the respective figure for 2021 was ~30%.
• Apart from the elderly, the magnitude of excess deaths was high in the young (aged 15-39), translating to a large burden of productive years lost. Over a quarter of life-years lost occurred during working age, pointing to economic and labour market consequences of excess mortality.
• Each excess death before retirement in Poland meant an average loss of 14–15 years of productive life.

## Introduction

The SARS-CoV-2 spread triggered an enormous health crisis worldwide, leading not only to increased mortality and morbidity but also profound social and economic consequences. As of 1 July 2025, 7.1 million deaths have been attributed to COVID-19 globally [1]. Yet, the pandemic’s death toll extends beyond confirmed infections, as numerous studies have established through an assessment of excess mortality [2–9]. While most of these studies focused on general epidemiological measures, such as death counts, mortality rates, and life expectancy losses, they often overlooked a comprehensive, age-specific analysis of premature mortality and how many lost years fell within the working age. To bridge this gap, we quantify Poland’s excess mortality burden during the COVID-19 pandemic and aftermath using two complementary metrics, Years of Potential Life Lost (YPLL) [10, 11] and Years of Potential Productive Life Lost (YPPLL) [12, 13]. While YPLL captures premature longevity losses before a determined threshold, YPPLL highlights economic impacts by accounting for years lost from deaths at working age solely. Both measures rest on the excess mortality framework but provide different policy focuses; YPLL refers to the population health dimension solely, whereas YPPLL signals human capital losses and reduced labour force participation, indicating macroeconomic implications of diseases [14]. Importantly, no prior study has measured productive life-years lost due to COVID-19 in Poland.

We base our YPLL and YPPLL estimates on the excess mortality framework–an extensively used approach that quantifies a surplus of deaths attributable to an extraordinary event, e.g. pandemic [3, 5, 8]. This allows us to compare our findings to previous empirical studies from Poland, which were focused on excess mortality [15, 16] and years of life lost [17], while extending the perspective to incorporate the productivity dimension of excess mortality.

Poland experienced a marked increase in all-cause deaths during the first two pandemic years. There were 64 thousand excess deaths in 2020 (15.5% more than expected under a non-pandemic scenario) [15] driving a 1.4-year drop in life expectancy, one of the largest declines in Europe [18]. Age-specific analyses have shown that mortality among middle-aged adults in Poland was disproportionately high [19], indicating a considerable loss of individuals at working age. This burden has broader economic implications. Even before the pandemic, Poland experienced notable labour shortages [20, 21] and population ageing [22, 23]. The COVID-19 crisis intensified structural demographic and labour challenges, contributing to reduced productivity and increased pressure on pension [24] and healthcare systems [25].

In this study, we enrich existing evidence on the excess mortality burden caused by the COVID-19 pandemic by contributing in three ways. First, we estimate both excess YPLL (eYPLL) and excess YPPLL (eYPPLL), moving beyond a purely epidemiological perspective to one that also captures economic burden. Second, we analyse five years (2020–2024), spanning both acute and post-pandemic phases, a timespan not yet covered in COVID-19 studies. Third, by calculating excess mortality in narrow, 5-year age groups, we gain a nuanced understanding of premature mortality patterns across different life course stages.

Therefore, we use pre-pandemic (2011–2019) weekly all-cause mortality data from Poland to forecast expected mortality for 2020–2024, and we derive excess death counts to convert them into eYPLL and eYPPLL. This dual-metric framework delivers a comprehensive picture of how COVID-19 reshaped Poland’s longevity profile and diminished its labour supply potential.

## Methods

### General approach

This study used country-level, sex- and age-group-specific data on all-cause mortality in Poland (2011–2024) to estimate the excess mortality burden during the pandemic and post-pandemic, capturing both longevity and productive-life losses. We applied three measures of excess mortality: eYPLL, eYPPLL, and excess deaths. The basis for the estimation of all three measures is the excess mortality concept, referring to a situation where observed mortality during unusual circumstances (e.g. pandemic, heatwave) exceeds mortality that would have been observed in the absence of these circumstances [26–28]. The weekly data on mortality rates from the baseline period of 2011–2019 were used to build a linear regression model with weekly fixed effects, which was then applied to predict expected mortality rates in the pandemic and post-pandemic years, 2020–2024. The choice of the baseline period was determined by data availability, and its length was similar to that used by a recent study from France [29]. The difference between observed and predicted mortality rates yielded excess mortality, which was used to determine excess death counts, eYPPL, and eYPPLL, as explained below.

Additionally, we analysed life-years lost measures (YPLL and YPPLL) using cause-specific mortality data; this was done by applying the following COVID-19-related ICD-10 codes: U07.1 (COVID-19, virus identified); U07.2 (COVID-19, virus not identified); and U10 (Multisystem inflammatory syndrome associated with COVID-19).

We used weekly death counts broken down by sex and 5-year age groups (0–4, 5–9, …, 80–84, 85+) published by Statistics Poland [30]. Statistics Poland reports time-related data using the ISO8601 standard, meaning that weeks are not comparable across particular calendar years in terms of their starting dates and length (most years consist of 52 weeks, but some [2015 and 2020 in the period analysed] had 53 weeks; also, 1 st week of a ISO8601 year usually does not start at 1 January and might contain days from previous calendar year). To increase comparability across years, we restructured data on death counts according to the approach applied by Hajdu et al. [31]. Using this adjusted dataset on death counts, we first calculated sex- and age-specific mortality rates per 100,000 population for each week and sex- and age-strata. For population data, we used counts on 31 December of each year from Statistics Poland’s demographic database [32] and linearly interpolated entries for each week between available data points.

**Fig. 1:**
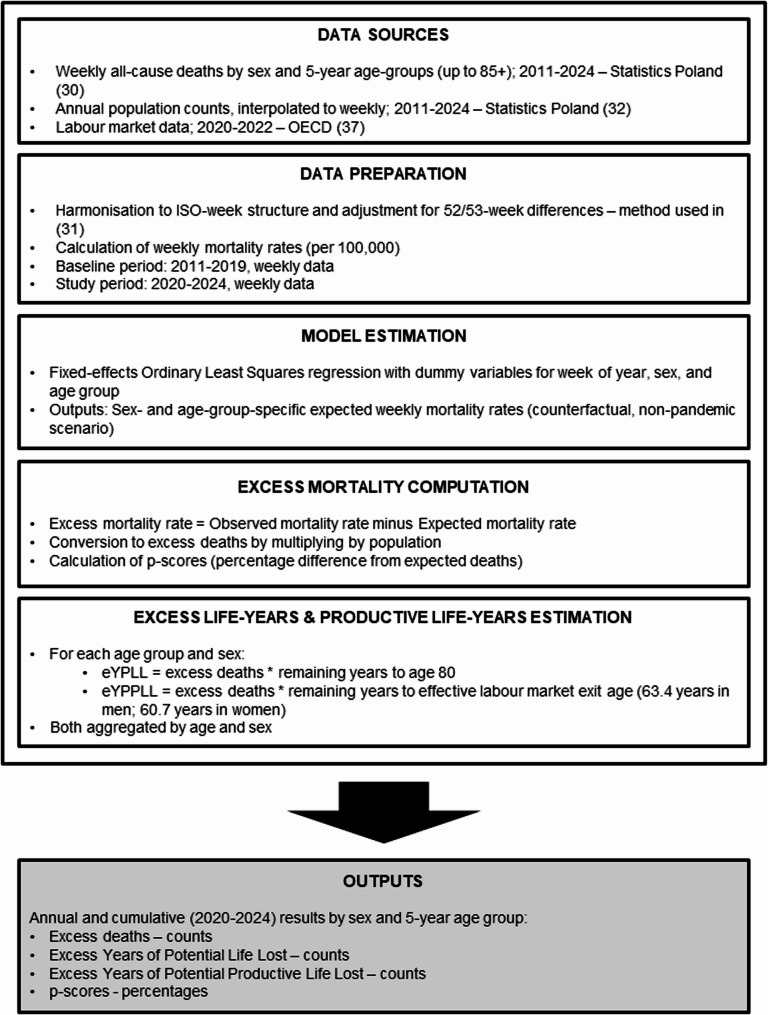
Overview of the analytical workflow for estimating excess deaths, eYPLL, and eYPPLL in Poland, 2020–2024

The overview of the analytical workflow used in this study is depicted in Fig. 1.

### Excess mortality and excess life-years lost estimation

To estimate excess mortality rates $\:{MR}_{i,s,a,t}$ for a particular week (*i*), sex (*s*), and a 5-year age group (*a*), we built a single fixed effects ordinary least squares (OLS) model with dummy variables for week of year, sex, and age group, similar to previous studies concerned with COVID-19 excess mortality [31, 33]. The following model was fitted for 2011–2019 data:

$$\begin{aligned}&{MR}_{i,s,a,t}=\:\beta\:+\:{\gamma\:}_{i}+\:{\delta\:}_{s}+\\&{\theta\:}_{a}+\:\mu\:t+\:{\epsilon\:}_{i,s,a}\:\mathrm{f}\mathrm{o}\mathrm{r}\:i=1,\\&\dots\:,52;t=2011,\dots\:,2019\end{aligned}$$

where: $\:\beta\:$ is an intercept term, $\:{\gamma\:}_{i}$ are week-of-year fixed effects capturing seasonality, $\:{\delta\:}_{s}$ are sex fixed effects, $\:{\theta\:}_{a}$ are age group fixed effects, $\:\mu\:$ is a coefficient of linear annual trend $\:t$, and $\:{\epsilon\:}_{i,s,a}$ is an error term. This model captures seasonal, age and sex variations and was used to project expected mortality rates for 2020–2024 in sex- and age-groups. The difference between observed and projected mortality rates yielded excess mortality rates, which were then multiplied by respective population counts to reach the number of excess deaths. Additionally, we also report excess mortality in terms of a p-score, a relative epidemiological measure recommended for reporting excess deaths; it shows the relative proportion of deaths due to the pandemic as the percentage of deaths above the expected level [34].

All the formulas used for estimating the above measures are presented in the Online Resource 1 (section A).

eYPLL was calculated as a product of annual excess deaths and duration of life from a mid-point of each 5-year age group in which a death occurred until the age of 80 years, e.g. for a death in age group 10–14, the number of years lost was 68 (80 minus 12) [10]. The sum of eYPLL for all age groups yielded the total eYPLL. The choice of 80 years as a cut-off point for YPLL estimation is consistent with COVID-19 studies that adopt this age to align with life expectancy in developed countries and to facilitate between-country comparability [10, 35, 36].

Likewise, we calculated eYPPLL similarly to eYPLL but replaced the duration of life with the duration of labour market activity in Poland, and the latter was calculated as the difference between the sex-specific average effective labour market exit age (average value for years 2020–2022; males: 63.4 years, females: 60.7 years) [37] and the age of starting the first regular job (20 for males and 21 for females [38]). Therefore, eYPPLL is a subset of eYPLL that lies within the working age.

The equations for calculating eYPLL and eYPPLL and shown in Online Resource 1 (section A).

## Results

### Mortality trends

**Fig. 2:**
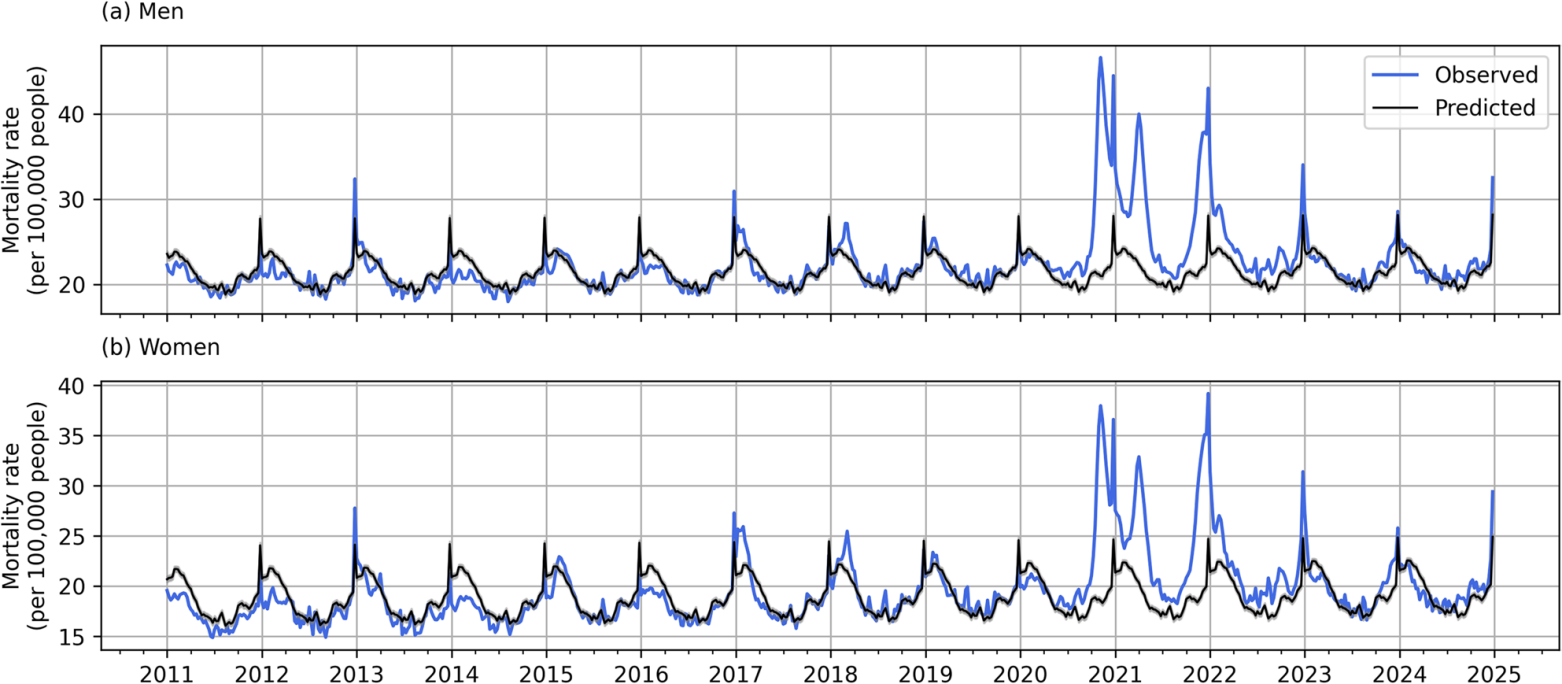
Observed and predicted mortality rates (deaths per 100,000 population) in Poland, 2011-2024. Notes: panel (**a**) - men, panel (**b**) - women.Source: own estimates based on Statistics Poland [30] data

Comparison of observed and expected mortality rates for men and women across the whole period exhibits notable deviations of observed values from the ones expected in the absence of the pandemic (Fig. 2). There was no severe excess mortality observed in Poland until the end of the third quarter of 2020. The highest deviations from expected mortality rates were identified in weeks 41–52 of 2020; weeks 12–17 of 2021; and weeks 45–52 of 2021. On the other hand, from the beginning of 2023, the values of expected and observed mortality follow similar paths with minor excess deviations in the last two quarters of 2023 and 2024, and minor reduced deviations in the first two quarters of these years.

### Excess deaths

Table 1 reports the number of excess deaths and p-scores for the five years analysed (2020–2024), both sexes, eighteen age groups and the all-age population. Altogether, there were 257,246 excess deaths in Poland in the 2020–2024 period, of which 138,229 were attributable to men (53.7%) and 119,017 to women (46.3%). These figures translate to 13.4% more deaths than expected in men and 12.2% more in women in the whole period, although p-scores for the first two pandemic years were much higher and exceeded 30% in 2021 among men.

**Table 1:** Table 1111. Excess mortality in Poland, 2020–2024: sex- and age-specific excess deaths and relative excess mortality (p-scores)

		Age-groups																		
		0–4	5–9	10–14	15–19	20–24	25–29	30–34	35–39	40–44	45–49	50–54	55–59	60–64	65–69	70–74	75–79	80–84	85+	total
Males																				
Excess deaths *p*-scores (%)	2020	−70* −7.8%	7 8.8%	−26* −20.9%	−9 −2.3%	96* 11.6%	76* 5.8%	122* 5.9%	469* 15.3%	648* 15.2%	734* 12.2%	1,255* 15.3%	1,007* 7.2%	2,667* 11.2%	5,144* 16.6%	7,424* 25.7%	4,683* 21.6%	6,196* 24.5%	11,368* 30.9%	41,796* 20.0%
	2021	12 1.4%	8 10.5%	−8 −6.4%	97* 26.7%	126* 16.4%	152* 12.2%	313* 15.6%	948* 31.7%	1,422* 34.2%	1,653* 27.3%	2,440* 30.5%	2,952* 22.4%	5,605* 24.8%	9,309* 30.1%	10,722* 35.4%	7,799* 35.7%	7,622* 31.5%	12,203* 34.2%	63,335* 30.8%
	2022	17 2.2%	17 23.3%	29* 24.3%	81* 23.2%	126* 17.5%	85* 7.2%	276* 14.1%	809* 28.0%	705* 17.4%	907* 15.0%	1,285* 16.3%	834* 6.6%	1,524* 7.1%	2,739* 8.8%	4,469* 14.4%	2,612* 11.1%	3,101* 13.4%	6,292* 17.8%	25,880* 12.7%
	2023	−15 −2.0%	19 27.9%	18 15.7%	91* 27.2%	87* 12.7%	36 3.2%	−34 −1.8%	479* 17.4%	628* 15.8%	366* 6.1%	434* 5.6%	−365* −3.0%	−647* −3.2%	−288 −0.9%	1,144* 3.6%	510 2.0%	681* 3.0%	1,790* 5.0%	4,988* 2.4%
	2024	−38 −5.7%	30* 48.2%	27 25.9%	131* 40.3%	56 8.7%	−54 −5.0%	32 1.7%	414* 15.9%	682* 17.5%	349* 5.9%	492* 6.3%	−294 −2.5%	−1,266* −6.5%	−1,103* −3.6%	1,218* 3.8%	70 0.2%	310 1.4%	1,107 3.0%	2,230 1.1%
	2020–2024	−94 −2.3%	81 22.4%	40 6.8%	391 22.2%	491 13.5%	294 4.9%	708 7.2%	3,119 21.8%	4,084 20.1%	4,009 13.3%	5,907 14.9%	4,134 6.5%	7,884 7.3%	15,801 10.2%	24,977 16.2%	15,674 12.9%	17,910 15.3%	32,760 18.2%	138,229 13.4%
Females																				
Excess deaths *p*-scores (%)	2020	−100* −13.6%	−14 −18.3%	−6 −5.8%	−2 −0.9%	46* 19.9%	15 4.6%	21 3.8%	169* 18.0%	148* 10.3%	98 4.4%	478* 15.3%	158 2.7%	654* 5.7%	1,693* 10.0%	3,918* 19.7%	3,452* 18.0%	4,881* 15.6%	15,252* 18.5%	30,866* 15.7%
	2021	−29 −4.0%	−5 −6.6%	10 10.1%	42* 24.3%	34* 15.2%	89* 29.7%	196* 37.4%	264* 28.8%	452* 31.9%	684* 30.3%	894* 29.4%	1,085* 19.4%	3,042* 27.8%	5,007* 29.4%	7,323* 34.8%	7,272* 38.0%	9,912* 33.2%	19,983* 24.5%	56,245* 28.9%
	2022	−34 −5.2%	9 12.6%	43* 46.1%	56* 32.4%	57* 26.1%	65* 22.6%	140* 27.3%	251* 28.2%	249* 17.9%	245* 10.8%	408* 13.7%	69 1.3%	705* 6.8%	831* 4.9%	2,457* 11.3%	2,993* 14.6%	5,381* 19.1%	11,202* 13.8%	25,112* 12.9%
	2023	−78* −12.8%	29* 40.2%	37* 41.3%	57* 32.7%	31* 14.8%	69* 25.0%	65* 13.1%	179* 21.2%	131* 9.5%	−29 −1.3%	280* 9.5%	−353* −6.8%	−295* −3.0%	−615* −3.6%	731* 3.3%	1,305* 5.9%	2,519* 9.5%	783 1.0%	4,878* 2.5%
	2024	−90* −16.2%	−10 −14.5%	23 27.1%	45* 25.1%	7 3.5%	1 0.5%	57* 11.9%	174* 21.6%	212* 15.8%	48 2.1%	125 4.3%	−424* −8.4%	−713* −7.6%	−1,077* −6.3%	97 0.4%	1,451* 6.0%	2,345* 9.2%	−414 −0.5%	1,916 1.0%
	2020–2024	−332 −10.1%	9 2.5%	107 23.2%	199 22.6%	176 16.0%	238 16.6%	479 18.7%	1,038 23.6%	1,192 17.1%	1,046 9.3%	2,186 14.5%	535 2.0%	3,394 6.5%	5,839 6.9%	14,526 13.5%	16,474 15.6%	25,038 17.7%	46,807 11.4%	119,017 12.2%

* - Statistically significant excess/reduced mortality. The aggregated 2020–2024 figures were not assessed for the significance of deviations from expected mortality levels. Confidence intervals for point estimates shown here are reported in Online Resource 1, table A1. Source: own estimates based on Statistics Poland [30] data

We observed consistent excess mortality for most of the years, age groups and both sexes. In general, the number of excess deaths increased with consecutive age groups; in men, it was < 100 among those aged 25–29 and below in most years and increased to > 11,000 in 2020 and 2021 in the oldest (85+). For women, < 100 excess deaths per year were observed up to the 30–34 age group in most years, while in the 85 + age group, the number of excess deaths reached almost 20,000 in 2021.

Almost half of all excess deaths (63,335 and 45.8% throughout the whole period in men; 56,245 and 47.3% in women) were identified in 2021, 25.9–30.2% in 2020, and around 1/5 in 2022. Of all the excess deaths throughout the five years, 1.6% occurred in 2024 (the same share for both sexes). For the age distribution of excess deaths, almost half of women’s excess deaths in 2020 (49.4%) were among the eldest (aged 85+). The respective share for the eldest men in the same year was much lower, 27.2%, and such a difference was sustained in 2021–2022 but not in 2023–2024, when the number of excess deaths declined notably. This sex difference illustrates the fact that men experienced excess deaths at younger ages than women did; e.g. for men aged 60–64 and 65–69 in 2020, the share of deaths in these age groups in total excess deaths was 6.4% and 12.3%, while the respective shares for women were only 2.1% and 5.5%. Again, this pattern was observed throughout the next two years (2021–2022) but not later.

Reduced mortality (negative value of excess deaths) was observed in some age groups and both sexes, but it was non-significant in several cases. However, we note that a significant reduction of mortality was identified in the young (aged 0–4 and 10–14 years) in 2020 and three age groups between 55 and 69 in 2023–2024.

Considering the relative burden of excess deaths, we note that the p-score values varied greatly across age groups, sexes and years. For most age groups and both sexes, it was 2021 when p-scores were the highest. In this year, there were >30% more deaths than expected among men aged 35–39, 40–44, 50–54, 65–69, 70–74, 75–79, 80–84 and 85+, and among women aged 30–34, 40–44, 45–49, 70–74, 75–79 and 80–84. Conversely, in the younger population, the highest p-score values were observed not in 2021–2022 but in 2023–2024 and exceeded 40% among those aged 5–9 and 10–14. For younger middle-aged men and women (aged 35–39 and 40–44), p-scores were consistently significant and above expected levels in each year. For two elderly age groups (aged 55–59 and 60–64), we identified negative p-scores in 2023–2024; expected mortality reduction in this group was higher in women than in men, reaching − 8.4% in women aged 55–59 in 2024.

**Fig. 3:**
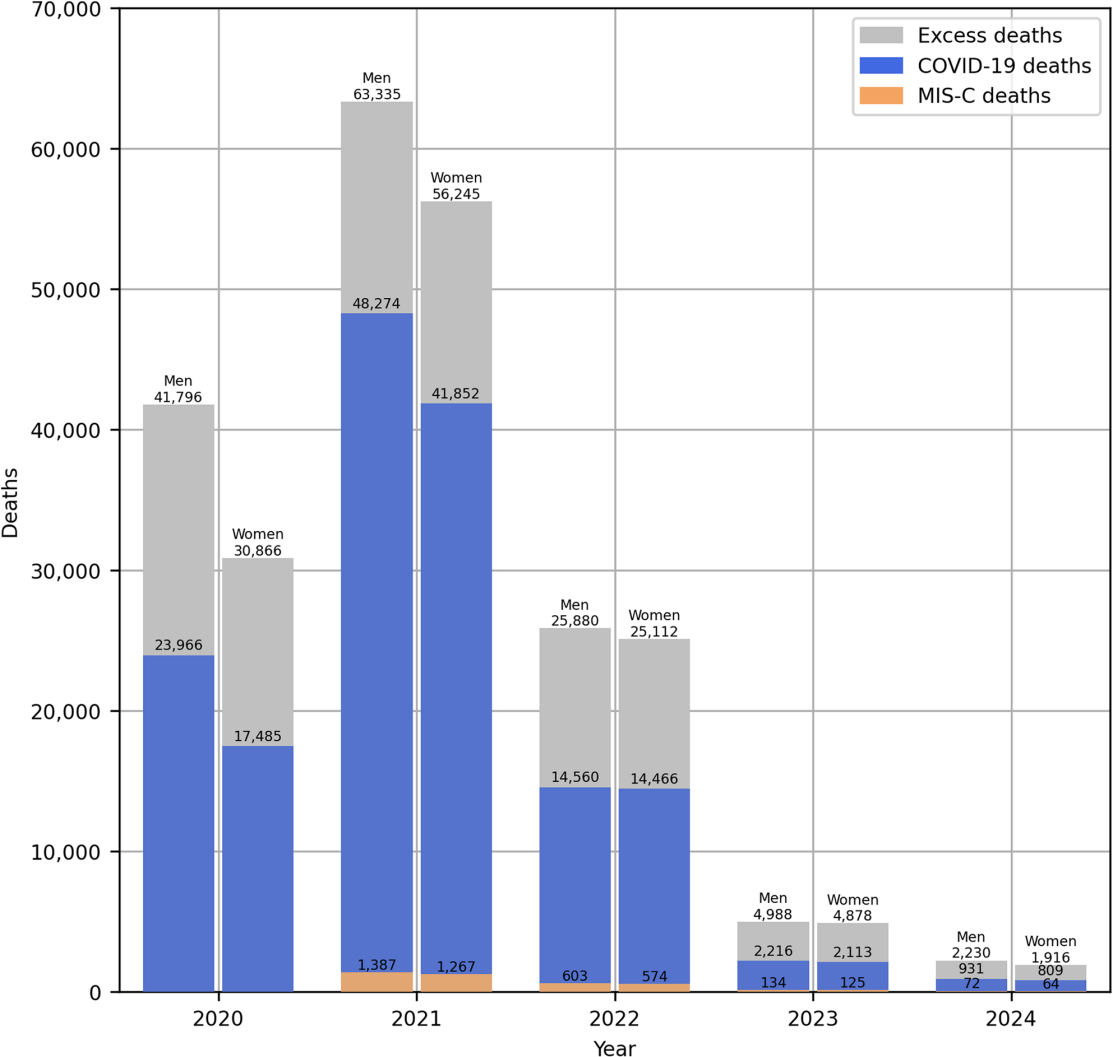
Excess deaths, COVID-19 deaths and multisystem inflammatory syndrome associated with COVID-19 deaths in Poland, 2020-2024. Source: own estimates based on Statistics Poland [30] data

Figure 3 compares annual excess deaths with deaths from COVID-19 (ICD-10 codes: U07.1 & U07.2) and from multisystem inflammatory syndrome associated with COVID-19 (MIS-C; U10) in 2020–2024. In the first pandemic year, 57% of excess deaths were caused by COVID-19 and this share was almost the same in 2022. In 2021, around 3/4 of all excess deaths were directly caused by COVID-19 and this share declined to 42–44% in 2023–2024. The deaths caused by MIS-C were first identified in 2021 and constituted 2.2%−3.3% of all excess deaths in the following years.

### Excess years of potential life lost

There were 1,921,265 eYPLL in Poland in 2020–2024 and 71.8% of these years were attributable to male mortality (1,380,244 in men and 541,021 in women; Table 2). The figure was 323,752 eYPLL in men in 2020, and it doubled in 2021 (654,154 eYPLL). In 2022, eYPLL returned slightly below the 2020 level and decreased greatly in the two following years. For women, eYPLL in 2020 was around a third of men’s level (103,777 eYPLL), but it increased sharply in 2021, tripling the initial level (318,161 eYPLL). In 2024, we identified negative eYPLL in women (−10,968) and this resulted from reduced mortality in three age groups below 80 years of age (a total of 2,213 reduced deaths in age groups 55–59, 60–64, and 65–69; Table 1).

**Table 2:** Table 2. Measures of years of potential life lost (YPLL) in Poland, 2020-2024

	2020	2021	2022	2023	2024	total
YPLL from excess deaths						
males	323,752	654,154	287,989	68,084	46,264	1,380,244
females	103,777	318,161	111,363	18,688	−10,968	541,021
males - per excess death	7.7	10.3	11.1	13.6	20.7	10.0
females - per excess death	3.4	5.7	4.4	3.8	−5.7	4.5
males - per 100,000 population	1,751	3,561	1,575	374	255	7,542
females - per 100,000 population	526	1,621	570	96	−56	2,765
YPLL from COVID-19 deaths^a^						
males	201,899	463,987	113,800	12,952	5,134	797,772
females	96,027	263,482	69,588	8,247	3,134	440,478
males - per COVID-19 death	8.4	9.3	7.5	5.5	5.1	8.8
females - per COVID-19 death	5.5	6.1	4.6	3.7	3.6	5.7
males - per 100,000 population	1,092	2,526	622	71	28	4,359
females - per 100,000 population	486	1,342	356	42	16	2,251

Notes: per death and per 100,000 population measures refer to the all-age population

^a^COVID-19 deaths here refer to both COVID-19 (ICD-10 codes: U07.1 & U07.2) and multisystem inflammatory syndrome associated with COVID-19 (U10). Source: own estimates based on Statistics Poland data [30].

The average YPLL per excess death in 2020 was 7.7 among men and 3.4 among women. In men, the figure almost doubled to 13.6 YPLL in 2023; in women, it peaked in 2021 with 5.7 YPLL. For 2024, the respective numbers are hardly comparable with previous years because in several 5-year age groups, we identified negative excess mortality, and this led to a relatively low, or even negative (for women), number of total eYPLL. Therefore, the values of 20.7 (men) and − 5.7 (women) YPLL per excess death should not be interpreted in the same way as the values from previous years. Considering eYPLL per 100,000 population, we identified that rates for women were around triple lower than the ones for men (e.g. 526 and 1,751, respectively, in 2020), apart from 2021, when the sex disparity was lower.

In the 2020–2024 period, around 2/3 (64.4%) of all eYPLL were attributable to COVID-19 deaths. In 2020, of the total 103,777 eYPLL in women, 96,027 (92.5%) were caused by COVID-19. The respective share among women declined in the following years to 82.8% in 2021 (263,482 of 318,161 eYPLL), 62.5% in 2022 (69,588 of 111,363 eYPLL) and 44.1% in 2023 (8,247 of 18,688 eYPLL) (it was not interpretable for 2024, when eYPLL in women was negative). For men, we identified a lower share of COVID-19-related YPLL in eYPLL; it was 62.4% in 2020 (201,899 of 323,752 eYPLL), increased to 70.9% in 2021 (463,987 of 654,154 eYPLL) and declined to 19.0% in 2023 (12,952 of 68,084 eYPLL) and 11.1% in 2024 (5,134 of 46,264 eYPLL). Additionally, in men, YPLL loss per death case was generally higher in excess deaths than in COVID-19 deaths, while for women, the opposite was true. For example, in 2021, the average loss of YPLL in the male population was 10.3 per excess deaths and 9.3 per COVID-19 deaths, while for women, the respective rates were 5.7 and 6.1 (Table 2).

### Excess years of potential productive life lost

There were 521,887 eYPPLL in Poland in 2020–2024, and 78.9% of these years were attributable to male mortality (411,742 in men and 110,145 in women). In 2020, we identified 69,058 eYPPLL among men; this number increased to 162,455 in 2021 and a year later was still higher than in 2020 (96,660 eYPPLL; Table 3). For women, the total eYPPLL was 4–6 times lower than for men in 2020 (10,921 compared to 69,058) and 2024 (8,939 and 41,040, respectively); in three other years, it was around three times lower for women and peaked in 2021 with 46,551 eYPPLL.

**Table 3:** Table 3. Measures of years of potential productive life lost (YPPLL) in Poland, 2020–2024

	2020	2021	2022	2023	2024	total
YPPLL from excess deaths						
males	69,058	162,455	96,660	42,529	41,040	411,742
females	10,921	46,551	29,093	14,641	8,939	110,145
males - per excess death	10.4	10.8	14.8	36.2	57.6	13.7
females - per excess death	9.9	11.2	17.6	38.7	129.4	15.0
males - per 100,000 population	444	1,056	635	282	275	2,703
females - per 100,000 population	76	329	207	105	65	783
YPPLL from COVID-19 deaths^a^						
males	35,877	90,893	22,836	1,876	681	152,164
females	11,678	32,929	9,435	1,023	387	55,452
males - per COVID-19 death	7.9	8.9	9.5	8.3	3.9	8.7
females - per COVID-19 death	9.3	9.9	10.7	12.6	6.6	9.9
males - per 100,000 population	231	591	150	12	5	999
females - per 100,000 population	82	232	67	7	3	394

Notes: per death and per 100,000 population measures refer to the pre-productive- and productive-age population (males: 0–63.4.4 years; females: 0–60.7.7 years)

^a^COVID-19 deaths here refer to both COVID-19 (ICD-10 codes: U07.1 & U07.2) and multisystem inflammatory syndrome associated with COVID-19 (U10). Source: own estimates based on Statistics Poland data [30].

The average YPPLL per excess death in 2020 was 10.4 among men and 9.9 among women and the figure increased consistently for the next two years, reaching 14.8 (men) and 17.6 (women) in 2022. For years 2023 and 2024, we observe high levels of YPPLL per excess death (> 35) due to large reduced mortality in some age groups; therefore, these rates should be treated with caution, similarly to the above eYPLL analysis. Considering eYPPLL per 100,000 population, we identified that rates for men were 2.7 to 5.8 times higher than in women, the greatest sex difference being recognised in 2020 (444 eYPPLL per 100,000 in men and 76 in women).

In the 2020–2024 period, 39.8% of all eYPPLL were attributable to COVID-19 deaths. This share was higher in the first two pandemic years; around half of the eYPPLL among men was caused by COVID-19 mortality. COVID-19-related male deaths led to 35,877 YPPLL in 2020 (90,893 in 2021), constituting 52.0% of 69,058 eYPPLL (56.0% of 162,455 in 2021) from excess deaths. In 2024, the share of eYPPLL in men caused by COVID-19 mortality decreased to 1.7% only (681 of 41,040 years lost). The respective shares were higher in females than in males; in 2020, the number of YPPLL attributable to COVID-19-related deaths (11,678) was higher than the eYPPLL (10,921). This means that in the female population, COVID-19 deaths disproportionately affected younger working-age populations compared to general excess deaths. However, in the following years, the number of YPPLL from COVID-19 was lower than the eYPPLL. In contrast to the overall eYPLL, the average loss of productive years was higher in excess mortality than in COVID-19 mortality.

### Age and sex distribution of eYPLL and eYPPLL

**Fig. 4:**
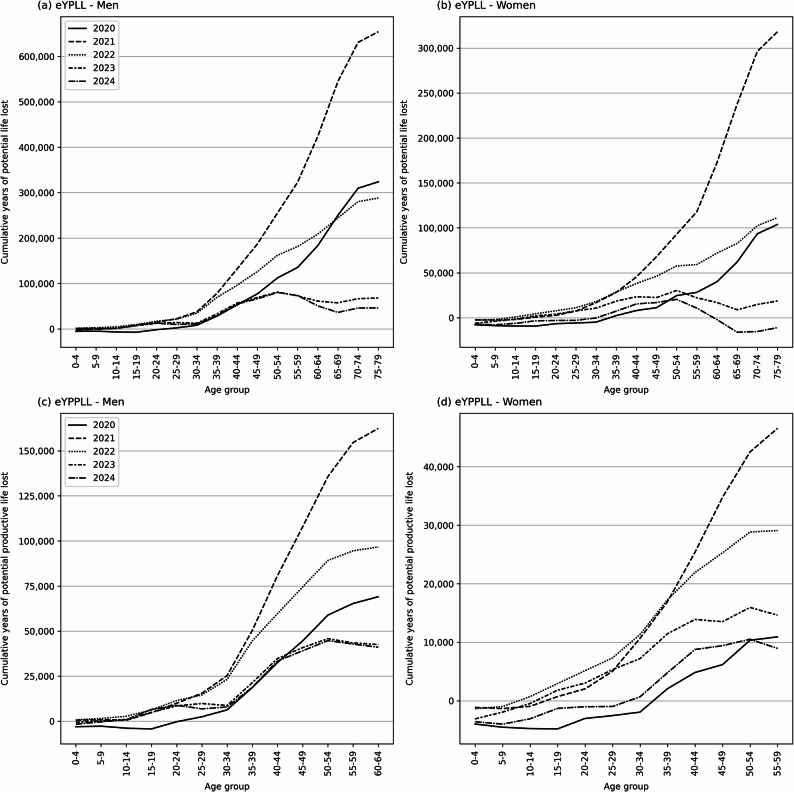
Cumulative excess years of potential life lost (eYPLL) and excess years of potential productive life lost (eYPPLL) by sex and age group in Poland, 2020–2024. Notes: panel (**a**) - eYPLL, men; panel (**b**) - eYPLL, women; panel (**c**) - eYPPLL - men; panel (**d**) - eYPPLL - women. Source: own estimates based on Statistics Poland [30] data

A closer examination of the age- and sex-specific distribution of eYPLL and eYPPLL reveals some further patterns. Across 2020–2024, over a quarter of all excess life-years lost (27% for both sexes, 30% in males and 20% in females) fell within working age, yielding 0.52 million eYPPLL out of 1.92 million eYPLL (Fig. 4; detailed data in Online Resource 1, tables A2 & A3). Age-sex patterns reveal interesting and sometimes diverging trends. First, cumulative curves for men steepen at the age of about a decade younger than the women’s; eYPLL builds rapidly from the 35–39 band for men but only from 45 to 49 for women, mirroring higher male mortality in mid-life. Second, a majority of productive years lost were identified in individuals aged 35–54; for women, the share of eYPPLL in this group was > 60% each year, while for men, > 50%. Third, cumulative eYPLL stops rising after 2022 and even turns negative in women aged 60 + in 2024, but no negative values appear for eYPPLL, because those elderly groups lie beyond retirement age. Finally, in the pandemic years (2020–2021), but also a transitional year 2022, the cumulative eYPLL rose steeply in older age groups. On the other hand, by 2023–2024, the longevity burden shifted from seniors toward middle-aged adults (declining cumulative eYPLL in those aged 50–69), underscoring that a sizeable share of lost life-years struck the labour force.

## Discussion

This is the first study using the excess mortality approach to estimate the socio-economic burden of mortality directly and indirectly attributable to the COVID-19 pandemic in Poland during the five years 2020–2024. Among other excess mortality measures, we analysed eYPPLL to demonstrate not only the pandemic’s epidemiological toll but also how the excess deaths translated to potential economic losses. We also contributed to the literature by providing the first estimates of excess mortality in the fifth year after the pandemic’s inception.

### Comparison to previous studies and interpretation of main findings

We identified 257,246 excess deaths in Poland in the 2020–2024 period, and this figure translates to 1,921,265 eYPLL of which more than a quarter (521,887) were productive years lost (eYPPLL). In the whole period, there were 13.4% more deaths than expected in men and 12.2% in women.

Considering excess deaths, our estimate is comparable to a recent study by Pizzato et al. [39], who estimated 223,735 excess deaths in Poland in 2020–2023, a timespan one year shorter than ours. Also, annual figures are similar in our study and [39], but only in the first two years (2020: 72,662 and 73,742; 2021: 119,580 and 117,525). In 2022, we identified 50,992 excess deaths, while the previous study – 39,683. The discrepancy between the two estimates was even higher for 2023; our estimate of 9,866 excess deaths was not comparable to the reduced (negative excess) mortality of −7,213 reported by Pizzato et al. [39]. This difference might arise from methodological choices; we used detailed 5-year age groups, while the study we compare to applied only five age intervals (0–14, 15–64, 65–74, 75–84 and 85+) [39]. The other studies on excess mortality in Poland are limited to the first two pandemic years and reported excess deaths of 64–69 thousand in 2020 [15, 40] and 157–214 thousand in 2020–2021, depending on the model specification used [2].

To our knowledge, there are no studies that estimated eYPLL or eYPPLL in Poland during the pandemic; however, we identified two studies using settings similar to ours. First, in their cross-country analysis of person-years of life lost across 18 European countries, Ahmadi-Abhari et al. [41] reported 2.52 million excess years of life lost in Poland through 2020–2022, whereas our corresponding eYPLL estimate is 1.80 million. These results are not directly comparable as [41] applied a life tables-based measure of remaining life expectancy, while we relied on the fixed upper-age cut-off of 80 years. Importantly, among 18 countries analysed, Poland ranked second in the rate of years lost per 1,000, only after Estonia and showed more than five-fold higher rate than Sweden, the country with the most favourable outcome [41]. Second, Orlewska et al. [17] estimated YPLL in Poland for 2020, but their analysis dealt with COVID-19 deaths, not excess mortality. Their estimates of local (country-specific) and standard (based on the Global Burden of Disease study) YPLL yielded losses of 436,361 and 630,027 years lost. We based our estimation on a different methodological approach [10] and used excess, not COVID-19, mortality measures; therefore, our estimate of 427,529 eYPLL in 2020 cannot be directly compared to those of [17]. However, we should note that our study found that YPLL attributable directly to COVID-19 in 2020 constituted 70% of eYPLL (Table 3); therefore, our estimates are relatively lower.

We found that almost half of all excess deaths in Poland occurred in 2021 alone, a time concentration characteristic of Central and Eastern European (CEE) states that recorded the highest excess mortality during that year. P-score in Poland in 2021 was 30.8% among men and 28.9% among women, exhibiting a massive over-mortality. Apart from our study, a similar time pattern was identified in a cross-country study [39] for Bulgaria, Czechia, Estonia, Croatia, Hungary, Lithuania, Latvia and Slovakia. Importantly, after a record-high mortality in 2021, CEE countries returned to a lower level of excess deaths in 2022, with declines of ~ 60% compared to 2021 excess mortality in Bulgaria, Czechia, Hungary, Poland and Slovakia [39]. Our findings are clearly in line with these results. However, several Western European countries exhibited a delayed excess mortality compared to CEE. France experienced the highest excess mortality in 2022 and a substantial excess death toll in 2023 [29]. The highest excess mortality of the period was identified in 2022 in Austria, Germany, Denmark, Finland, Iceland, and Norway [39]. This East-West discrepancy in the timing of the mortality burden distribution can be attributed to many factors, including the severity of restrictions applied and differences in vaccination uptake. Poland introduced strict non-pharmaceutical interventions at the pandemic’s inception [42–44] and initially avoided a large mortality burden; however, the country experienced a major increase in excess deaths in late 2020 and 2021 [16], due to slower vaccine uptake (as of 1 Jan 2023, only 56.8% of the Polish population was fully vaccinated, while in the European Union this share was 72.8% [45]) and challenges in sustaining public health measures [44, 46]. Consequently, relatively low excess mortality in 2022–2023 in Poland (but also in other CEE countries, as shown in [39]) plausibly arises from a harvesting effect, a compensatory phenomenon resulting from the depletion of the vulnerable population in the previous course of the pandemic [47, 48].

Our results indicate that the majority of excess deaths in Poland were directly linked to COVID-19; e.g. around three-quarters of excess deaths in the most lethal year (2021) carried a U07 code. In the remaining years, this share ranged from 42% to 58%, illustrating that indirect pandemic effects also contributed substantially to overall over-mortality. As established in epidemiological literature, such indirect effects stem from health system disruptions that restricted access to timely diagnosis and treatment, as well as behavioural factors, such as vaccination uptake, delayed health-seeking, or adherence to non-pharmaceutical interventions [7, 9, 49]. Nevertheless, accurate identification of COVID-19 deaths remains challenging, and the proportion of deaths directly caused by the virus may be biased downward due to limited testing capacity in some periods.

### Sex and age disparities in excess mortality burden

Several studies have found that COVID-19 has been more lethal for men [50] and older individuals [51]. Our results confirm these findings; however, considering age-specific mortality, we also identified notable excess deaths in the younger population.

53.7% of excess deaths in the whole period were attributable to male mortality, but this share was the highest in 2020 (57.5%), and it generally declined with the course of the pandemic. Therefore, men seemed to be more prone to excessive deaths initially, but later on, the sex distribution of mortality became more proportional (50.6% of male excess deaths in 2023), possibly suggesting an earlier occurrence of the harvesting effect in men, as shown in other settings [52, 53]. Compared to the number of excess deaths, the sex difference is much more pronounced in potential years of life lost, particularly in productive ages. 71.8% of the whole-period eYPLL was due to men’s mortality, and the respective share for eYPPLL was 78.9%. The former figure shows that men’s premature deaths were concentrated in younger age groups than in women, while the latter additionally highlights the difference in the duration of economic activity. As men work longer on average, their death at a certain age represents a higher potential productive loss than women’s death at the same age [38].

An important finding from our research is a substantial excess mortality in the young population. For those aged 15–19, we identified relative excess mortality (p-score) of 23%−40%, depending on year and sex (apart from 2020). Slightly lower p-scores, but still exhibiting notable excess deaths, were also estimated for other young adult groups and both sexes. For example, 2,370 men aged 35–44 died in excess in 2021, and this corresponded to >30% p-score. For women of the same age, the number of excess deaths was lower (716), but in relative terms, it was still around 30% more than the expected level. This pattern is consistent with findings from Hungary, where the highest rate ratio for excess death in the third pandemic wave (weeks 7–21 of 2021) was identified in the population aged 35–44 [54]. On the other hand, in England, the young (aged 20–44) were most burdened in terms of excess mortality in 2022 [55]. The high excess mortality in the young population might originate from non-biological factors, e.g. lower adherence to non-pharmaceutical interventions in this group [54] or increased alcohol-related mortality during the pandemic [56, 57].

P-scores comparable to those in the young were identified only among the population aged 70+. The highest relative excess mortality in both sexes was found in 2021 among those aged 70–74 (men: 35.4%; women: 34.8%) and 75–79 (men: 35.7%; women: 38.0%).

Our analysis reveals interesting findings regarding negative excess, in other words, reduced mortality. First, we identified a lower mortality level than expected in the youngest group (aged 0–4) in some years. Several prior studies pointed to decreased mortality in infants and children and attributed this to fewer infections and accidents during lockdowns [58, 59]. Our findings show that the lower-than-expected mortality in this group was sustained also post-pandemic, and mortality was reduced by a higher share in 2024 than in 2020 (in women). This might reflect behavioural changes (better hygiene, stay‑home‑while‑sick norms) and new ways of protection that became more prevalent during and after the pandemic, e.g. universal rotavirus immunisation and maternal COVID‑19 boosters that reduce infant health risks [60, 61].

Additionally, the post-pandemic (2023–2024) reduced mortality was observed in three age groups between 55 and 69, and for both sexes. For women, this reduction was more pronounced and reached a p-score of −8.4% in the 55–59 group in 2024 (for men, the highest reduction of −6.5% in the 60–64 group in 2024). This favourable pattern likely reflects a combination of interrelated factors. First, the harvesting effect (also referred to as mortality displacement) may have occurred, as numerous frail individuals died earlier in the course of the pandemic, and this affected population structure by temporarily reducing the vulnerable population in subsequent years [47, 52]. Second, a post-pandemic rebound in the health system’s capacity to meet routine care needs that are crucial for survival (e.g. diagnostic and elective treatments) [25] may have reduced mortality among older adults. Finally, early-pandemic behavioural changes such as improved hygiene, remote work, and reduced mobility may have had lasting protective effects [62, 63].

### Economic impact of excess productive-life years lost

Our study adds to the current knowledge on pandemic-related excess mortality by quantifying eYPPLL, an economic dimension that most epidemiological studies did not account for. Until 2024, Poland had lost 521,887 years of productive life more than expected in the absence of the pandemic, and 40% of these losses resulted from deaths in 2021 solely. The impact of losses was heavily skewed toward men; their total eYPPLL were four to six times higher than women’s in 2020 and 2024, and three times higher in 2021–2023. This gap stems from higher male mortality, the fact that male deaths clustered at younger working ages and their longer work activity (effective retirement age of 63.4 vs. 60.7 in women).

The eYPPLL estimates show that not only were many lives lost, but a notable fraction of this loss (27% throughout the whole period) hit people in their productive years, amplifying economic and social disruption. Each death at a young- or mid-age removes more than a decade of productive life (13.7 productive years lost in men and 15.0 in women; Table 3), depleting human capital that would otherwise contribute to socio-economic welfare. The economic consequences of the mortality surge are numerous: increased pre-existing labour shortages (Poland has had one of the lowest unemployment rates across EU countries), productivity challenges, and increased strain on social systems due to fewer workers and taxpayers but more widows and orphans to support [64, 65]. All these factors potentially threaten economic growth and long-term welfare. Compared to Western European countries, Poland did not avoid a shift of mortality burden onto younger generations [66] and this translates to large eYPPLL that might restrict potential economic output in future.

Our findings carry important policy implications for labour market planning and healthcare prioritisation. The notable reduction of labour supply resulting from premature mortality calls for coordinated policies that enhance workforce participation and strengthen rehabilitation programmes, facilitating return to work, particularly among individuals with chronic conditions whose health deteriorated during the pandemic. From the healthcare perspective, the identified over-mortality among the working-aged underscores the need to improve preventive and primary care and ensure equitable access to rehabilitation services [25]. Integrating detailed mortality surveillance with labour and health policies could help anticipate productivity losses and guide resource allocation to mitigate long-term economic consequences of future health crises. Such actions are particularly important for countries facing labour supply shortages, such as Poland [21].

Although this study did not translate the labour supply reductions into output terms, we can provide a rough estimate of the broader macroeconomic consequences of COVID-19-related premature mortality in Poland. With a productive age population of around 23.3 million, 521,887 eYPPLL correspond to ~ 0.45% of reduced potential labour input during 2020–2024. Accounting for the average employment rate among those 15–64 in the period (71.1%), and using the OECD production function approach (where output elasticity of labour is 0.67) [67], the eYPPLL identified would imply a gross domestic product decline of roughly 0.21%. This is a conservative estimate of potential losses as it does not account for morbidity-related labour reductions. Additionally, it should be verified with a formal analysis of productivity losses in monetary terms using the human capital approach [68].

An additional consequence of the pandemic arises from the decreased productivity of COVID-19 survivors. Several studies have found that the history of novel coronavirus infection deteriorates labour outcomes [69, 70]. Because of the mortality focus of this study, we were unable to investigate the economic burden from morbidity; yet, this topic requires research to supplement mortality-based evidence.

### Study strengths and limitations

To our knowledge, this is the first study to estimate excess mortality over a 5-year horizon (2020–2024). Previous studies, in particular the ones focusing on Poland, were limited to the first pandemic year or two and did not account for the post-pandemic endemic phase, which also requires follow-up [2]. Additionally, by estimating eYPPLL, we examined the pandemic-attributable mortality burden in terms of its socioeconomic magnitude. To do so, we used detailed age- and sex-specific models that allowed us to disentangle mortality patterns in narrow 5-year age groups. Eventually, the source data we used is considerably reliable, as Poland’s death registration system is assessed to be complete or nearly complete according to international evaluation [71] and Statistics Poland provides comprehensive coverage of all registered death cases.

Despite these contributions, we shall note the following caveats of our analysis. First, estimation of excess mortality is subject to numerous, often subjective, methodological choices, including the length of period used to build a baseline model, estimation technique, data aggregation (e.g. in terms of age bin width) and the choice of mortality measure (counts or rates), among others. Previous studies have shown [26, 72] that these choices might lead to results that differ notably. However, these estimates’ differences should rather be seen as providing complementary, not contradictory, evidence. Second, we predicted expected mortality up to five years ahead (until 2024) and this raises questions about the reliability of such long predictions. We aimed to tackle this issue by securing a longer-than-usual baseline period (2011–2019, while most studies focus on five pre-pandemic years), but this approach has its limitations. Third, as for eYPLL and eYPPLL estimation, a standard approach to deal with these measures does not account for the underlying conditions of those dying prematurely, and this fact plausibly overestimates years lost due to the pandemic (for more details, see [29]). Our approach, following the cross-country study [10] uses a threshold of 80 years for both sexes and might bias our estimates upwards in men and downward in women compared to life expectancy in Poland. Fourth, to estimate eYPLL and eYPPLL, we modelled excess mortality in 5-year age bins, and such models might be less reliable for the young, where the number of deaths is lower than in the elderly. Fifth, COVID-19 deaths are known to be under-ascertained in mortality statistics in many countries. Although our all-cause excess mortality framework is robust to such under-coding, the attribution shares we present (e.g. the share of COVID-19 deaths in excess deaths) are affected by this limitation and may be lower than the true values. Poland has been recognised as a country with relatively low testing capability [18]; therefore, COVID-19-coded shares of excess mortality should be interpreted as lower-bound estimates. Additionally, estimates of the excess deaths fraction directly caused by COVID-19 may be biased by temporal differences in testing capacity and by early misclassification, as some COVID-19 deaths were likely coded under respiratory diseases before the U07 code was introduced. Sixth, the study design does not warrant a causal effect of the pandemic on mortality. Eventually, we did not account for other determinants that are known to affect mortality, e.g. socioeconomic factors (income level and distribution, access to health care) that changed independently of COVID-19 spread but might have also affected population health.

## Conclusions

This study provided a comprehensive picture of excess mortality patterns in Poland during the COVID-19 pandemic and post-pandemic period (2020–2024). We achieved this by combining excess death counts with excess life years and productive life years lost. More than 257 thousand excess deaths in the country translated into 1.9 million life-years lost, 27% of which fell within the productive age. This shows that COVID-19 has affected not only the elderly population, but also those at their prime working age, removing an average of 14–15 productive years for every excess death before retirement. This pandemic-borne loss of labour capacity magnifies Poland’s pre-existing worker shortage, slows productivity growth and increases pressure on the country’s economy.

## Supplementary Information

Supplementary Material 1.

## Data Availability

The authors confirm that all data generated or analysed during this study can be replicated with publicly available sources as reported in the Methods section.

## Abbreviations

CEE: Central & Eastern Europe
eYPLL: Excess Years of Potential Life Lost
eYPPLL: Excess Years of Potential Productive Life Lost
OECD: Organisation for Economic Co–operation and Development
OLS: Ordinary Least Squares
YPLL: Years of Potential Life Lost
YPPLL: Years of Potential Productive Life Lost
