# Supplementary material for: Excess life-years and productive life-years lost in Poland through the COVID-19 pandemic and post-pandemic years
Source: Arch Public Health. 2026 Jan 15;84:11. doi: 10.1186/s13690-025-01792-0 (PMC12805778; doi:10.1186/s13690-025-01792-0)
Supplement: Supplementary file 1 — Supplementary Material 1. [file 13690_2025_1792_MOESM1_ESM.pdf]

Online Resource 1

Article title: Excess life-years and productive life-years lost in Poland through the COVID-19 pandemic and post-pandemic years

Journal name: Archives of Public Health

Author names: Błażej Łyszczarz, Jakub Wojtasik, Tomasz Zieliński

Affiliation and e-mail address of the corresponding author: Nicolaus Copernicus University in Toruń, Department of Health Economics, Bydgoszcz, Poland; blazej@cm.umk.pl

A. Detailed description of excess mortality, eYPLL, and eYPPLL estimation

Excess mortality

To estimate excess mortality rates $MR_{i,s,a,t}$ for a particular week (*i*), sex (*s*), and a 5-year age group (*a*), we built a single fixed effects ordinary least squares (OLS) model with dummy variables for week of year, sex, and age group. The following model was fitted for 2011-2019 data:

$MR_{i,s,a,t} = \beta + \gamma_i + \delta_s + \theta_a + \mu t + \varepsilon_{i,s,a}$ for $i = 1, \ldots, 52; t = 2011, \ldots, 2019$

where: β is an intercept term, γ_i are week-of-year fixed effects capturing seasonality, δ_s are sex fixed effects, θ_a are age group fixed effects, μ is a coefficient of linear annual trend t, and $\varepsilon_{i,s,a}$ is an error term.

Subsequently, for each i, s, a and $\hat{t} \in 2020 - 2024$ we computed predicted mortality rate $\widehat{MR}_{i,s,a,t}$ as:

$\widehat{MR}_{i,s,a,t} = \beta + \gamma_i + \delta_s + \theta_a + \mu\hat{t}$

Computed age-specific mortality rates were used to determine all-age estimates of the mortality across sexes:

$$\widehat{MR}_{i,s,t} = \frac{\sum_{a \epsilon A}(\widehat{MR}_{i,s,a,t} \times Pop_{i,s,a,t})}{Pop_{i,s,t}}$$

Because these estimates are constructed as weighted sums of linear models, they remain linear models themselves, allowing us to estimate the coefficients and report weekly 95% confidence intervals.

Furthermore, yearly mortality rates were computed using estimators of sex-specific mortality rate models:

$$\widehat{MR}_{s,t} = \frac{\sum_{i=1}^{52} \widehat{MR}_{i,s,t} \times Pop_{i,s,t})}{Pop_{s,t}}$$

Yearly confidence intervals were calculated independently using the formula:

$$CI_{s,t} = \left[\widehat{MR}_{s,t} - \boldsymbol{t}_{1-\frac{\alpha}{2}, n-p} \cdot \hat{\sigma} \cdot \sqrt{m + 1^T X^* (X^T X)^{-1} X^{*T} 1}, \widehat{MR}_{s,t} + \boldsymbol{t}_{1-\frac{\alpha}{2}, n-p} \cdot \hat{\sigma} \cdot \sqrt{m + 1^T X^* (X^T X)^{-1} X^{*T} 1}\right],$$

where:

X – matrix of observations used for building $\widehat{MR}_{i,s,a,t}$ estimators,

X^* – matrix of predicted (2020-2024) observations,

X^T– transposition of matrix,

X^{-1} – inverse of nonsingular matrix,

m – number of predicted (2020-2024) observations (5 years × 52 weeks = 260),

$\hat{\sigma}$ – square root of unbiased estimator of variance of random errors in the final model (enabling to compute values of $\widehat{MR}_{i,s,t}$),

$\mathbf{1}$ – vector of ones (of appropriate size),

$\boldsymbol{t}_{1-\frac{\alpha}{s},n-p}$ – the $(1-\frac{\alpha}{2})$ quantile of the Students's t-distribution with $(n-p)$ degrees of freedom,

n – number of rows in X,

p – number of columns in X,

α – significance level.

Eventually, the excess mortality rate $eMR_{i,s,a,t}$ was defined as the difference between observed and predicted mortality rates:

$$eMR_{i,s,a,t} = MR_{i,s,a,t} - \widehat{MR}_{i,s,a,t}$$

Next, we multiplied sex- and age-specific excess mortality rates $eMR_{i,s,a,t}$ and population counts $Pop_{i,s,a,t}$ to reach numbers of excess deaths in each stratum:

$$eDeaths_{i,s.a.t} = eMR_{i,s,a,t} \times Pop_{i,s,a,t}$$

We also report excess mortality in terms of a p-score, a relative epidemiological measure recommended for reporting excess deaths; it shows the relative proportion of deaths due to the pandemic as the percentage of deaths above the expected level:

$$p - score = \frac{oDeaths_{i,s,a,t} - eDeaths_{i,s,a,t}}{eDeaths_{i,s,a,t}}$$

where $oDeaths_{i,s,a,t}$ is an observed number of deaths.

Accordingly, all the excess mortality measures for all-age and year aggregations were calculated using the approach described above for mortality estimation.

Excess years of potential life lost (eYPLL)

The formula used for eYPLL is given by:

$$eYPLL_{s,t} = \sum_{i}\sum_{a}(eDeaths_{i,s,a,t} \times L_a)$$

where L_a are the remaining years of life for age group *a* up to age 80, capped at zero for age groups with midpoints ≥80:

$L_a = \max(0, 80 - m_a)$ with m_a being a midpoint of age group *a* (e.g., 12 for 10–14).

Excess years of potential productive life lost (eYPPLL)

The formula used for eYPPLL is given by:

$$eYPPLL_{s,t} = \sum_{i}\sum_{a}(eDeaths_{i,s,a,t} \times L_a^{prod})$$

where L_a^{prod} are remaining productive years of life for age group *a* defined as:

$$L_a^{prod} = \begin{cases} A_{exit} - A_{entry}, \text{if } m_a < A_{entry} \\ \max(0, A_{exit} - m_a), \text{if } m_a \in [A_{entry}, A_{exit}) \\ 0, \text{otherwise} \end{cases}$$

A_{entry} being the age of starting the first regular job (20 for males and 21 for females) and A_{exit} – effective labour market exit age (63.4 for males, and 60.7 years for females).

Table A1. Sex- and age-specific excess deaths in Poland, 2020-2024: point estimates (confidence intervals)

MALES

Year/Age	0-4	5-9	10-14	15-19	20-24	25-29	30-34	35-39	40-44	45-49
2020	-70<br>(-133; -7)	7<br>(-13; 28)	-26<br>(-51; -2)	-9<br>(-52; 35)	96<br>(36; 155)	76<br>(5; 146)	122<br>(30; 214)	469<br>(338; 600)	648<br>(496; 799)	734<br>(541; 928)
2021	12<br>(-54; 77)	8<br>(-14; 30)	-8<br>(-34; 18)	97<br>(52; 143)	126<br>(65; 187)	152<br>(81; 223)	313<br>(218; 407)	948<br>(810; 1,086)	1,422<br>(1,260; 1,583)	1,653<br>(1,441; 1,865)
2022	17<br>(-49; 84)	17<br>(-7; 40)	29<br>(1; 57)	81<br>(31; 130)	126<br>(64; 189)	85<br>(13; 156)	276<br>(179; 374)	809<br>(665; 953)	705<br>(532; 877)	907<br>(675; 1,140)
2023	-15<br>(-81; 52)	19<br>(-6; 44)	18<br>(-12; 47)	91<br>(37; 146)	87<br>(22; 152)	36<br>(-37; 109)	-34<br>(-135; 66)	479<br>(331; 627)	628<br>(442; 815)	366<br>(113; 619)
2024	-38<br>(-104; 27)	30<br>(4; 57)	27<br>(-3; 58)	131<br>(70; 191)	56<br>(-12; 124)	-54<br>(-128; 20)	32<br>(-72; 135)	414<br>(262; 566)	682<br>(481; 882)	349<br>(73; 624)
Total	-94	81	40	391	491	294	708	3,119	4,084	4,009

Year/Age	50-54	55-59	60-64	65-69	70-74	75-79	80-84	85+	Total
2020	1,255<br>(1,027; 1,483)	1,007<br>(725; 1,288)	2,667<br>(2,226; 3,109)	5,144<br>(4,557; 5,731)	7,424<br>(6,859; 7,989)	4,683<br>(4,209; 5,157)	6,196<br>(5,621; 6,771)	11,368<br>(10,440; 12,297)	41,796<br>(38,356; 45,235)
2021	2,440<br>(2,198; 2,683)	2,952<br>(2,663; 3,240)	5,605<br>(5,156; 6,055)	9,309<br>(8,683; 9,935)	10,722<br>(10,087; 11,357)	7,799<br>(7,286; 8,312)	7,622<br>(7,032; 8,212)	12,203<br>(11,240; 13,166)	63,335<br>(59,719; 66,952)
2022	1,285<br>(1,022; 1,549)	834<br>(534; 1,134)	1,524<br>(1,065; 1,982)	2,739<br>(2,070; 3,408)	4,469<br>(3,769; 5,168)	2,612<br>(2,016; 3,207)	3,101<br>(2,493; 3,708)	6,292<br>(5,272; 7,313)	25,880<br>(22,059; 29,701)
2023	434<br>(146; 723)	-365<br>(-680; -50)	-647<br>(-1,115; -179)	-288<br>(-1,004; 429)	1,144<br>(377; 1,912)	510<br>(-198; 1,218)	681<br>(47; 1,314)	1,790<br>(683; 2,896)	4,988<br>(943; 9,034)
2024	492<br>(174; 810)	-294<br>(-626; 39)	-1,266<br>(-1,745; -786)	-1,103<br>(-1,866; -341)	1,218<br>(379; 2,056)	70<br>(-769; 910)	310<br>(-358; 979)	1,107<br>(-102; 2,316)	2,230<br>(-2,054; 6,514)
Total	5,907	4,134	7,884	15,801	24,977	15,674	17,910	32,760	138,17

FEMALES

Year/Age	0-4	5-9	10-14	15-19	20-24	25-29	30-34	35-39	40-44	45-49
2020	-100<br>(-155; -45)	-14<br>(-31; 3)	-6<br>(-28; 17)	-2<br>(-26; 23)	46<br>(17; 75)	15<br>(-18; 47)	21<br>(-23; 65)	169<br>(108; 230)	148<br>(65; 231)	98<br>(-8; 204)
2021	-29<br>(-86; 28)	-5<br>(-23; 13)	10<br>(-15; 34)	42<br>(16; 68)	34<br>(5; 63)	89<br>(57; 122)	196<br>(151; 242)	264<br>(200; 329)	452<br>(364; 540)	684<br>(567; 800)
2022	-34<br>(-92; 23)	9<br>(-10; 29)	43<br>(17; 69)	56<br>(28; 84)	57<br>(27; 87)	65<br>(32; 98)	140<br>(99; 186)	251<br>(183; 318)	249<br>(155; 343)	245<br>(118; 373)
2023	-78<br>(-136; -21)	29<br>(9; 50)	37<br>(10; 64)	57<br>(26; 88)	31<br>(0; 63)	69<br>(35; 102)	65<br>(17; 114)	179<br>(110; 249)	131<br>(29; 232)	-29<br>(-168; 111)
2024	-90<br>(-147; -33)	-10<br>(-32; 11)	23<br>(-6; 51)	45<br>(10; 79)	7<br>(-25; 40)	1<br>(-33; 35)	57<br>(8; 107)	174<br>(103; 245)	212<br>(103; 321)	48<br>(-103; 199)
Total	-332	9	107	199	176	238	479	1,038	1,192	1,046

Year/Age	50-54	55-59	60-64	65-69	70-74	75-79	80-84	85+	Total
2020	478<br>(355; 601)	158<br>(-13; 329)	654<br>(398; 910)	1,693<br>(1,327; 2,059)	3,918<br>(3,479; 4,356)	3,452<br>(3,005; 3,900)	4,881<br>(4,143; 5,620)	15,252<br>(13,188; 17,317)	30,866<br>(26,763; 34,968)
2021	894<br>(762; 1,025)	1,085<br>(911; 1,260)	3,042<br>(2,781; 3,303)	5,007<br>(4,617; 5,396)	7,323<br>(6,829; 7,816)	7,272<br>(6,791; 7,753)	9,912<br>(9,153; 10,671)	19,983<br>(17,817; 22,150)	56,245<br>(51,929; 60,561)
2022	408<br>(266; 551)	69<br>(-112; 250)	705<br>(439; 970)	831<br>(416; 1,246)	2,457<br>(1,914; 3,001)	2,993<br>(2,441; 3,545)	5,381<br>(4,604; 6,158)	11,202<br>(8,901; 13,504)	25,112<br>(20,549; 29,676)
2023	280<br>(124; 436)	-353<br>(-542; -163)	-295<br>(-564; -25)	-615<br>(-1,057; -173)	731<br>(136; 1,325)	1,305<br>(657; 1,954)	2,519<br>(1,719; 3,318)	783<br>(-1,696; 3,262)	4,878<br>(44; 9,713)
2024	125<br>(-46; 297)	-424<br>(-623; -224)	-713<br>(-987; -439)	-1,077<br>(-1,545; -608)	97<br>(-549; 744)	1,451<br>(691; 2,212)	2,345<br>(1,516; 3,175)	-414<br>(-3,102; 2,274)	1,916<br>(-3,206; 7,037)
Total	2,186	535	3,394	5,839	14,526	16,474	25,038	46,807	119,017

Table A2. Sex- and age-specific excess Years of Potential Life Lost (eYPLL) in Poland, 2020-2024: point estimates

MALES

Year/Age	0-4	5-9	10-14	15-19	20-24	25-29	30-34	35-39	40-44	45-49
2020	-5,491	529	-1,787	-563	5,555	4,019	5,856	20,172	24,613	24,229
2021	934	592	-534	6,129	7,316	8,056	15,015	40,775	54,023	54,546
2022	1,365	1,228	1,959	5,091	7,322	4,490	13,254	34,775	26,780	29,943
2023	-1,134	1,377	1,199	5,756	5,031	1,889	-1,656	20,596	23,882	12,080
2024	-2,976	2,197	1,853	8,238	3,241	-2,861	1,519	17,799	25,908	11,505
Total	-7,303	5,922	2,690	24,652	28,464	15,593	33,989	134,118	155,206	132,303

Year/Age	50-54	55-59	60-64	65-69	70-74	75-79	Total
2020	35,139	23,155	48,012	66,871	59,393	14,049	323,752
2021	68,334	67,886	100,891	121,019	85,775	23,396	654,154
2022	35,981	19,187	27,426	35,606	35,749	7,835	287,989
2023	12,160	-8,403	-11,639	-3,738	9,154	1,530	68,084
2024	13,768	-6,754	-22,781	-14,343	9,741	211	46,264
Total	165,383	95,071	141,908	205,415	199,813	47,021	1,380,244

FEMALES

Year/Age	0-4	5-9	10-14	15-19	20-24	25-29	30-34	35-39	40-44	45-49
2020	-7,803	-1,015	-387	-95	2,671	774	998	7,263	5,625	3,235
2021	-2,245	-360	661	2,643	1,975	4,726	9,428	11,372	17,177	22,560
2022	-2,683	680	2,925	3,529	3,306	3,434	6,698	10,774	9,460	8,098
2023	-6,098	2,140	2,508	3,615	1,825	3,633	3,135	7,717	4,965	-946
2024	-7,036	-758	1,552	2,832	427	68	2,744	7,489	8,056	1,583
Total	-25,865	686	7,259	12,523	10,204	12,636	23,003	44,615	45,283	34,531

Year/Age	50-54	55-59	60-64	65-69	70-74	75-79	Total
2020	13,390	3,633	11,780	22,011	31,341	10,356	103,777
2021	25,028	24,962	54,750	65,086	58,582	21,816	318,161
2022	11,435	1,579	12,685	10,805	19,660	8,979	111,363
2023	7,847	-8,114	-5,304	-7,996	5,844	3,916	18,688
2024	3,511	-9,745	-12,828	-13,998	780	4,354	-10,968
Total	61,211	12,315	61,083	75,908	116,207	49,422	541,021

Table A3. Sex- and age-specific excess Years of Potential Productive Life Lost (eYPPLL) in Poland, 2020-2024: point estimates

MALES

Year/Age	0-4	5-9	10-14	15-19	20-24	25-29	30-34	35-39	40-44	45-49
2020	-3,055	314	-1,141	-388	3,965	2,760	3,831	12,385	13,861	12,041
2021	520	352	-341	4,222	5,222	5,533	9,822	25,034	30,423	27,108
2022	759	730	1,250	3,507	5,226	3,084	8,670	21,351	15,081	14,881
2023	-631	819	765	3,966	3,591	1,297	-1,083	12,645	13,449	6,003
2024	-1,656	1,306	1,183	5,675	2,313	-1,965	994	10,928	14,590	5,718
Total	-4,063	3,521	1,717	16,982	20,317	10,709	22,234	82,342	87,406	65,750

Year/Age	50-54	55-59	60-64	Total
2020	14,307	6,443	3,734	69,058
2021	27,822	18,890	7,847	162,455
2022	14,649	5,339	2,133	96,660
2023	4,951	-2,338	-905	42,529
2024	5,606	-1,879	-1,772	41,040
Total	67,334	26,455	11,037	411,742

FEMALES

Year/Age	0-4	5-9	10-14	15-19	20-24	25-29	30-34	35-39	40-44	45-49
2020	-3,972	-552	-226	-60	1,782	492	596	4,003	2,768	1,343
2021	-1,142	-196	386	1,666	1,318	3,005	5,637	6,268	8,453	9,366
2022	-1,366	370	1,708	2,224	2,206	2,184	4,005	5,938	4,655	3,362
2023	-3,104	1,164	1,464	2,278	1,218	2,310	1,874	4,253	2,444	-393
2024	-3,581	-412	906	1,785	285	43	1,641	4,128	3,965	657
Total	-13,165	373	4,238	7,892	6,809	8,034	13,754	24,590	22,284	14,335

Year/Age	50-54	55-59	Total
2020	4,161	584	10,921
2021	7,776	4,016	46,551
2022	3,553	254	29,093
2023	2,438	-1,305	14,641
2024	1,091	-1,568	8,939
Total	19,019	1,981	110,145
